# Rethinking the process of detrainment: jets in obstructed natural flows

**DOI:** 10.1038/srep39103

**Published:** 2016-12-15

**Authors:** Michele Mossa, Francesca De Serio

**Affiliations:** 1 Technical University of Bari, Dicatech, Department of Civil, Environmental, Building Engineering and Chemistry, Via E. Orabona 4, 70125 Bari, Italy

## Abstract

A thorough understanding of the mixing and diffusion of turbulent jets released in porous obstructions is still lacking in literature. This issue is undoubtedly of interest because it is not strictly limited to vegetated flows, but also includes outflows which come from different sources and which spread among oyster or wind farms, as well as aerial pesticide treatments sprayed onto orchards. The aim of the present research is to analyze this process from a theoretical point of view. Specifically, by examining the entrainment coefficient, it is deduced that the presence of a canopy prevents a momentum jet from having an entrainment process, but rather promotes its detrainment. In nature, detrainment is usually associated with buoyancy-driven flows, such as plumes or density currents flowing in a stratified environment. The present study proves that detrainment occurs also when a momentum-driven jet is issued in a not-stratified obstructed current, such as a vegetated flow.

The interaction of aquatic vegetation and, more generally, of porous obstructions in natural environments with a stream flow remains insufficiently explored and represents a challenge for scientists. In fact, multiple ecological, morphological and physical aspects characterizing plant canopies, such as shoot density, leaf length, plant stiffness, standing biomass, as well as plant submergence ratio, size and location, influence the flow hydrodynamics^1^,^2^. On the other hand, hydrodynamics affect the structure of the underwater vegetation fields by dispersing spores, by mediating the availability of nutrients^2^,^3^, and by exerting drag forces and associated turbulence^4^,^5^, which could be responsible for vegetation development and survival^6^.

There is a continuous and mutual interaction between the canopy and its surrounding flow. It is well known that seagrasses are able to significantly influence the hydrodynamic environment by reducing current velocity, dissipating wave energy and increasing deposition or retention of finer sediments, to which benthic invertebrates are sensitive^7^,^8^,^9^,^10^. They have important ecological consequences, reducing turbidity and increasing light penetration, thus enhancing primary production and photosynthesis, which in turn guarantee their growth and reproduction.

Therefore, as also pointed out by Nepf^11^, aquatic vegetation is of tremendous significance to many ecosystem functions: (i) promoting biodiversity and creating different habitats with spatial heterogeneity in the stream velocity; (ii) improving water quality due to the uptake of nutrients and production of oxygen^12^; (iii) reducing coastal erosion and enhancing bank stability, as in the case of marshes mangroves and riparian vegetation^13^.

For all the above mentioned reasons, vegetation hydrodynamics should not be strictly studied from a hydraulic perspective. Nepf^11^ observed that the presence of vegetation modifies the velocity field across several scales, relevant to different processes. For example, the uptake of nutrients by an individual blade depends on the boundary layer on that blade, i.e. on the blade-scale flow. Similarly, the capture of pollen is mediated by the flow structure generated around individual stigma^14^. On the contrary, the retention or release of organic matter, sediments, seeds and pollen from a meadow or patch depends on the flow structure at the meadow or patch scale^15^. Furthermore, spatial heterogeneity in the canopy-scale parameters and architecturally varying components may originate complex flow patterns, which is difficult to interpret, even if using data collected from real channels with live vegetation.

In this extremely complex context, the current research^16^,^17^, carried out at the DICATECh of the Technical University of Bari (Italy), adds a turbulent jet as a new element interacting with a streamflow through a canopy. The aim is to provide a more thorough understanding of the hydrodynamic behavior of jets released in obstructed channels. In fact, it is worth to noting that vegetated channel flows have been studied in depth by many researchers^11^,^18^,^19^,^20^. Because of their practical applications, ranging from effluents discharged in atmosphere and water bodies, to combustion and thrust control, also turbulent jets have been widely analytically, computationally and experimentally studied^16^,^21^,^22^,^23^,^24^.

Nevertheless, studies on turbulent jets interacting with porous obstructions are still rare in literature. Moreover, the issue can also be of interest for its applicability not strictly limited to vegetated flows (Fig. 1a), but extended to a wider range of real cases. In fact, we can consider that also the planting of random or regular arrays of trees, used as protection and management systems of floodplains and banks, as well as solar power plants, offshore wind farms^25^ and oyster or mussel farms (Fig. 1b) could be generally considered as canopies. Hence, the release of water of boats between oyster farms, the airflow produced by spraying during pesticide treatments on orchards, submarine pollination can be considered as typical examples of this process.

In this paper, firstly a thorough study of the theory governing the phenomenon is carried out, secondly, some experimental results of momentum jets issued in a vegetated flow are examined. The theoretical study highlights an innovative aspect in the jet diffusion process, due to the interaction with the canopy. In fact, jet dynamics are generally associated with the entrainment process, i.e. the one-way transport process from the ambient fluid to the flowing turbulent fluid. As pointed out by Mc Climans^26^, the entrainment process was defined in pioneering studies as the erosion by turbulence of surrounding non-turbulent fluid. It can be intended as the net transport of fluid from a less to a more turbulent fluid, considering that turbulence intensity can be conceived as a measurement of turbulence.

Nevertheless, in some specific configurations, jets experience detrainment, which is the process by which fluid is expelled from a turbulent flow. Detrainment is an important mechanism in some fluid flows in nature, often observed in cloud dynamics, when cloudy air is transferred outside of the cloud volumes, with consequent influence on the vertical heat and moisture fluxes in the atmosphere^27^. Also in the ocean, detrainment is an important process, which explains the sediment transport from large density currents on the continental slope as well as the fate of hydrothermal plumes from submarine volcanic rift zones in the middle of the ocean^27^.

In any case, to the authors knowledge, previous studies point out that detrainment can occur when a jet or a plume impinges on a stratified interface^23^,^27^,^28^,^29^, without investigating into the necessary conditions for detrainment. Previous research studies^27^,^30^ analyzed plumes and thermals in a uniform environment, observing that a type of detrainment could be produced by buoyancy reversal, affecting the trajectory of the flow. It should be remarked that transport across a stratified interface is an essential aspect of many geophysical processes, such as pollution transport and dispersion by winds in urban areas or dispersion of effluents from sewage in coastal waters (Fig. 2). For this reason, to quantify the physical processes occurring at the interface, a profound understanding of turbulent mixing, entrainment and vortex dynamics at the interface has always been pursued, also considering that the more accurate these deductions are, the more transport models can be developed accurately^27^.

In any case, all these previous research studies principally focused on the entrainment rate or the behavior of turbulence at the interface, rather than on the conditions for detrainment. This brief review shows that the physics of jet detrainment is still not fully understood, and even its occurrence in different flows is an unresolved question. While detrainment in nature is usually associated with buoyancy-driven flows, like plumes or density currents flowing in a stratified environment, the present study demonstrates that detrainment occurs also when a momentum-driven jet is issued in a not-stratified obstructed current, such as a vegetated flow.

## Theoretical Framework and Results

**Figure 3:**
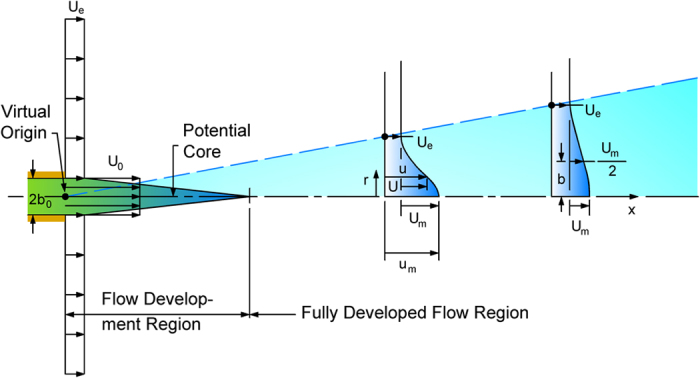
Sketch of a compound jet.

Considering that generally jets are issued in an ambient fluid with the presence of currents^31^, without loss of generality in terms of effects of the vegetation on the jets, below we will consider the case of a turbulent jet with a circular nozzle whose fluid is discharged into a large body of water with equal density, i.e. no buoyancy effects are present. Considering Fig. 3, the diameter of the jet at the exit is equal to *D* = 2*b*_*0*_ and the exit uniform velocity is equal to *U*_*0*_. The ambient flow velocity is *U*_*e*_. As well-known, the fully developed flow starts downstream of the core region, whose length has an order of magnitude of 12*b*_*0*_. Below we will consider only the fully developed region, where the longitudinal velocity distribution in each cross section, i.e. the distribution of the *u*-velocity in the radial direction, has the same well-known Gaussian shape shown in Fig. 3. At each cross section the maximum longitudinal velocity component is *u*_*m*_ and *b* is the typical length scale, which is generally assumed as the distance from the jet centerline where the longitudinal velocity is *u* = *u*_*m*_*/2*.

In the present paper we consider the case of jets issued in an ambient flow with a periodic square array of cylinders of uniform diameter *d* and distance *s*. Other key parameters of the cylinder array used in the present paper are the solid volume fraction *ϕ*, i.e. the volume within the canopy occupied by solid elements, which is the complement of the canopy porosity *1* − *ϕ*, the frontal area per unit volume of the canopy *a* = *nd*, which is equal to *d/s*^*2*^ in the case of a periodic square array, where *n* is the number of elements per unit planar area. For the sake of brevity, the theoretical development will report only some equations. Appendix 1 shows the complete theoretical development of a plane turbulent jet issued in a still obstructed fluid. The case of a compound circular jet has been preferred in the present analysis because, even if more complex from a theoretical point of view, generally it is much more common in nature. In any case, the mathematical development of a circular compound jet starts from the analysis of the simpler case of a plane turbulent jet in still water. Therefore, Appendix 1 enables the reader to better understand that reported below.

Within the array the flow is spatially heterogeneous at the scale of the individual elements and often unsteady in time. Generally, in the case of obstructed flows the double-averaging method is used to remove the temporal and element-scale spatial heterogeneity of the current^32^,^33^. In other words, the instantaneous equations of the vegetated current are first averaged over a time longer than the time scale of turbulence or instabilities in the flow and then averaged over an infinitesimally thin area that spans many cylinders, including only the area occupied by the fluid. In the case of the present study, only the time-average operator will be used, since a spatial average of the jet flow would lack information on the typical spatial variations of the jet longitudinal and transversal sections. For the sake of simplicity, the ambient current *U*_*e*_ is considered uniform.

Using cylindrical coordinates, the motion equation in the longitudinal direction *x* is


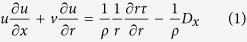


where (referring to Fig. 3)


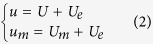


*τ* being the turbulent shear stress and *D*_*x*_ the drag force in the *x* direction, i.e. the resistance due to the solid medium, sum of form and viscous drag over the stem. As shown by Nepf^34^, various resistance laws for flows in porous media can be derived. Particularly, in open channel or atmospheric vegetated flow, the quadratic form is used, which, in the case of a compound turbulent circular jet, can be written using local velocity scales as follows


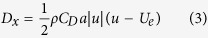


Koch and Ladd^35^ verified that a quadratic drag law describes the resistance at moderate-to-high Reynolds numbers in cylinder arrays.

Integrating along the radial direction the motion eq. (1) and using eq. (3) for the drag force, we get


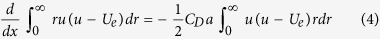


assuming that *C*_*D*_*a* is constant with the radial direction. This hypothesis is rigorous only in the case of obstructions formed by circular stems concentric with the jet axis, which generally is not common in nature. Nevertheless, the assumed hypothesis is reasonable considering the stem configuration, the ratios between the jet scale (*b*), the stem diameter (*d*) and distance (*s*) which will be considered and shown below in the present paper. The integral of the left side of eq. (4) is the integral momentum of a compound pure circular jet. Therefore, eq. (4) shows an interesting result, since in an obstructed flow the integral momentum of the jet is not preserved along the longitudinal direction *x*, as in the analogous case of an unobstructed flow, when *C*_*D*_ is nil.

For the similarity analysis, we can write


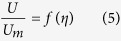


being *η* = *r/b.*

Rewriting eq. (4), with consideration of eqs (2) and (5), we get


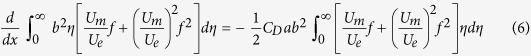


The similarity analysis allows to write also


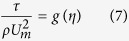


Therefore, assuming


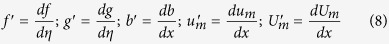


and inserting eqs (5), (7) and (8) in eq. (1), the motion equation becomes


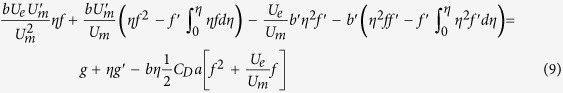


Furthermore, we can assume simple forms for *U*_*m*_ and *b*, such as:


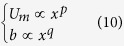


### The case of strong jets

Referring to the case of strong jets, i.e. *U*_*e*_*/U*_*m*_ ≪ 1, eq. (6) can be simplified and becomes


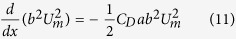


Therefore, taking into account eq. (10), we derive


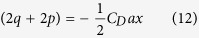


In the same hypothesis of strong jets, eq. (9) can be simplified too, providing


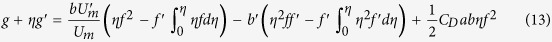


Since the left hand side of eq. (13) is independent of *x*, the same must be for the right hand side, i.e.


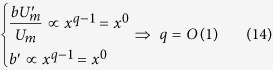


In order to consider the last term of the right side of eq. (13) independent of *x*, some considerations are reported below. For eqs (10) and (14) we can write that


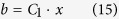


In the present study, some simplifications can be done, considering that we refer to jets with


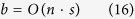


where *n* = *O*(10–100).

**Figure 4:**
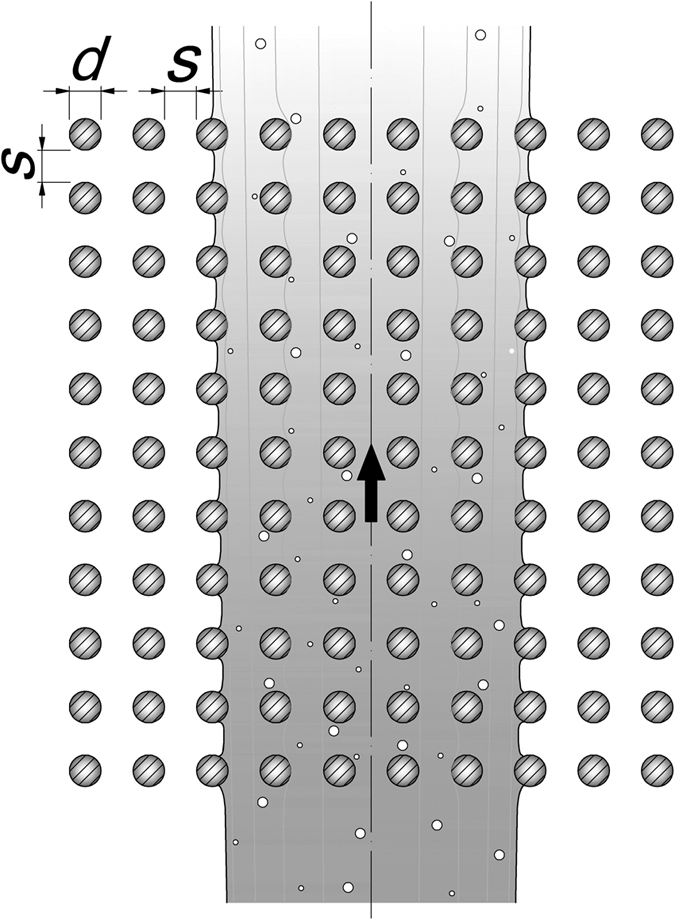
Sketch of the investigated jet and canopy geometry (top view).

In other words we are taking into account the case most frequently observed in nature, where *d/s* = *O*(10^−1^–1), *b/d* = *O*(10–10^2^), *b/s* = *O*(10–10^2^), as shown in Fig. 4.

Therefore, with the above mentioned orders of magnitude, it is possible to write that, if


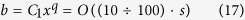


the order of magnitude of *b* changes when it becomes


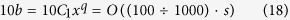


i.e. when *x*^*q*^increases of an order of magnitude or more. Therefore, *b* has the same order of magnitude between two values of *x*, i.e. from *x*_*1*_ to *x*_*2*_ > *x*_*1*_, when


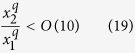


Since in the analyzed case *q* = 1, as derived from eq. (14), it is possible to write that


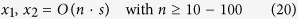


Therefore, along a longitudinal distance between *x*_*1*_ and *x*_*2*_ satisfying eq. (19), the last term of eq. (13) can be considered approximately constant in the limits above described.

Therefore, using eq. (12) we finally get


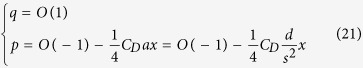


### The case of weak jets

When the jets are weak, i.e. *U*_*e*_*/U*_*m*_ ≫ 1, eq. (9) can be simplified and rewritten as follows


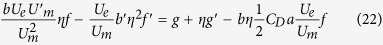


The first two terms of the right side of eq. (22) are independent of *x*. In order to consider also the last term of the right side of eq. (22) independent of *x* we can assume *b* with the same order of magnitude between two values of *x*, i.e. from *x*_*1*_ to *x*_*2*_ > *x*_*1*_, for which eq. (19) is verified.

Furthermore, assuming


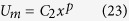


it is possible to assert that, considering two distance *x*_*1*_ and *x*_*2*_ > *x*_*1*_, the order of magnitude of *U*_*m*_ does not change if


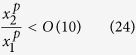


Considering the most restrictive hypothesis between (19) and (24), it is possible to conclude that, assuming that the right side of the eq. (22) is independent of *x*, also the left side must be independent of *x*.

In order to consider the left side of eq. (22) independent of *x*, we have


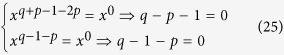


Furthermore, eq. (6) becomes


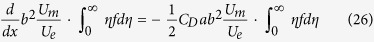


Therefore, eq. (26) can be written as follows


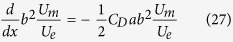


Considering eq. (8), from eq. (27) we can obtain


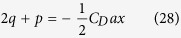


Therefore, using eqs (25) and (28) we get


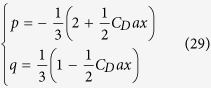


For the intermediate case of jets with *U*_*m*_*/U*_*e*_ ≈ 1 it is not possible to disregard some terms of the derived equations, therefore a simple exponential relation, as in the cases analyzed above, cannot be deduced.

### Entrainment coefficient analysis

Assume


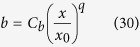



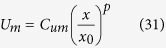


where *x*_*0*_ is the distance from the nozzle where the jet flow starts to be fully developed and *C*_*b*_ and *C*_*um*_ are the values of *b* and *U*_*m*_, respectively, for *x* equal to *x*_*0*_.

In the case of strong compound circular jets, the flow rate becomes equal to


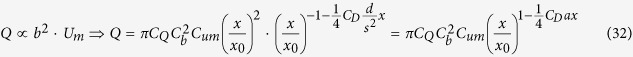


where *C*_*Q*_ *i*s a dimensionless coefficient which takes into account both the geometry of each cross section of the jet and the ratio between the average longitudinal velocity *U*_*b*_ and the maximum longitudinal velocity of each analyzed cross section. In other words, it is possible to write


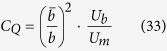


where 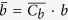 is the nominal outer boundary of the jet where *u* is close to *U*_*e*_. Therefore, we assume the hypothesis that the jet cross sections preserve their original circular shape, which of course is rigorous only in the case of obstructions formed by circular stems concentric with the jet axis. The assumed hypothesis is generally reasonable and, in any case, it is always possible to consider a rectified circular cross section equivalent to the real cross section.

The variation of the jet flow rate along the longitudinal axes is equal to


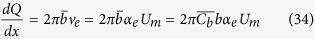


where *α*_*e*_ is the entrainment coefficient, i.e. the ratio between the velocity of the entrained fluid *v*_*e*_ and *U*_*m*_, with *v*_*e*_ the velocity transversal to the nominal outer boundary of the jet, which is oriented towards the jet centerline in the case of a positive entrainment coefficient and vice versa in the case of a negative entrainment coefficient, i.e. when a detrainment flow is present.

Considering eq. (34) and the derivative of eq. (32)


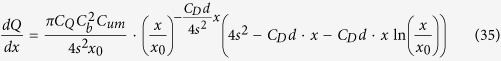


we get the following equation for the entrainment coefficient


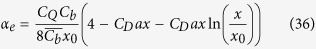


A different result is obtained in the case of weak compound jet, where


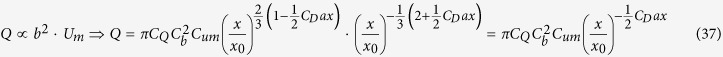


Therefore


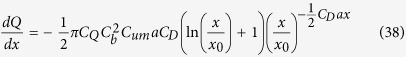


Considering eqs (34) and (38), it is possible to obtain the following equation of the entrainment coefficient


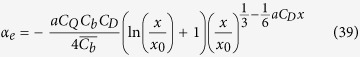


It is possible to highlight that in the case of a weak compound jet the variation of the jet flow rate is null as well as the entrainment coefficient, as shown by eq. (39), when obstructions are absent, i.e. with a nil bulk drag coefficient *C*_*D*_. On the contrary, in the presence of obstructions, the jet experiences a detrainment process as shown by eq. (39).

## Method

### Experimental procedure

The experimental runs were carried out in a smooth horizontal rectangular channel in the hydraulic laboratory of the DICATECh of the Technical University of Bari (Italy). The channel was 25.0 m long, 0.40 m wide and 0.50 m deep. The lateral walls and the bottom surface of the channel were constructed of Plexiglas. The outlet and the inlet structures of the channel were connected to a hydraulic circuit, allowing a continuous re-circulation of steady discharges. The channel was equipped with a series of stilling grids and a side-reservoir spillway with adjustable height in order to maintain a constant and uniform water head. In addition, it was equipped with two movable gates, placed at the inlet and the outlet of the channel, in order to regulate the channel flow rate and tailwater. At the downstream end of the channel, the water flow was intercepted by a rectangular reservoir, which was 3 m long, 1 m wide and 1 m deep, equipped with a V-notch sharp crested weir, in order to measure the channel flow rate. For further details on the experimental apparatus, please refer to Ben Meftah *et al*.^17^.

To simulate vegetation stems, a square array of rigid circular steel cylinders was used. The stem diameter, *d*, was equal to 0.003 m. The stems were inserted into a plywood board 3.0 m long, 0.398 m wide and 0.02 m thick, which in turn was fixed along the channel bottom, forming the experimental area. In order to reduce the effect of plywood board thickness on the experimental area, two other 3.0 m × 0.398 m × 0.02 m plywood boards, without vegetation stems, were attached to the channel bottom at both the upstream and the downstream side of this area. Stems were spaced longitudinally and transversally, with the same distance *s* = 0.05 m, so that the stem density was 400 stems/m^2^ and the vegetation density per meter was *a* = *nd* = *dH/s*^*2*^*H* = *d/s*^*2*^ = 1.2 m^−1^, with *ϕ* = *nπd*^*2*^*/4* = 0.00283 and *n* = 1− *ϕ*  = 0.997.

The jet source was placed at the center of the experimental area, 15.0 m and 0.2 m from the inlet and the side-walls of the channel, respectively. It consisted of a circular metallic pipe with a diameter, *D*, of 0.003 m. The jet was connected to a rectangular fiberglass tank by means of a plastic pipe. The tank was 1.0 m long, 0.5 m wide and 0.5 m deep and was positioned at a height of 3.6 m over the channel bottom surface. In order to maintain the jet discharge constant, the water flow was pumped continuously into the fiberglass tank by an electro-pump with a discharge larger than that of the jet. The water excess, distributed by the side-tank spillway, was driven via a pipeline to re-reach the reservoir from where the electro-pump absorbed the water.

The instantaneous three velocity components were measured accurately using a 3D Nortek Acoustic Doppler Velocimeter (ADV) system. The ADV was used with a velocity range equal to ±0.30 m/s, a velocity accuracy of ±1%, a sampling rate of 25 Hz and a sampling volume of 27 mm^3^.

## Discussion

The main characteristics of the analyzed runs are shown in Table 1, where *U*_*0*_ is the initial jet velocity, *Re* is the channel Reynolds number and *Re*_*0*_ is the initial jet Reynolds number. Therefore, both channel and jet flows were turbulent. The set of experiments were limited to the case of weak jets, because of peculiar interest for many natural cases, such as the pollen jets released into the wind. Each run was performed twice, firstly without obstructions and successively with obstructions. The geometry of the obstructions remained unchanged for all runs.

As shown by Nepf^34^, the bulk drag coefficient *C*_*D*_ is a function of the array density and, in the case of the present experiments, it was assumed equal to 1.2.

**Figure 5:**
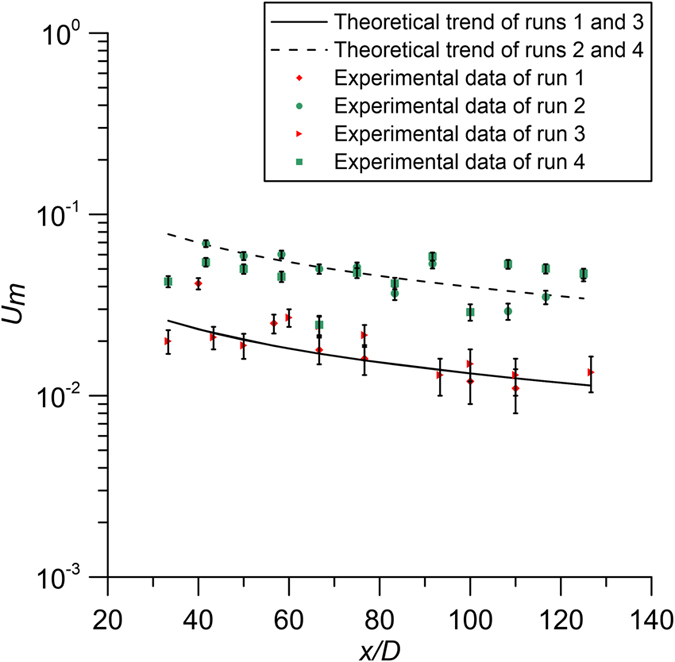
Experimental data of the velocity *U*_*m*_ superimposed to the theoretical trend.

Figure 5 shows the experimental values of *U*_*m*_ along the channel, compared with the theoretical law of eq. (31), for the case of weak jets, with the error bars. Some points of the diagrams are outside of the error bars, because of local velocity variations from the theory due to the relative distance of the measurement points from the stems. Considering all these aspects, we can conclude that the model presented in this paper can satisfactorily reproduce the flow field and, furthermore, we can infer other conclusions on the jet entrainment or detrainment. The analysis of Fig. 5 allows us to deduce that the coefficient *C*_*um*_ of eq. (31) is not a universal constant, changing with the velocity of the ambient fluid, and therefore with its Reynolds number.

The entrainment process can be defined as a one-way transport process from the ambient fluid to the flowing turbulent fluid of a jet. The implication of the entrainment process is an increased volume flux, which has to be taken into account in the continuity equation for volume. Of course, the opposite process occurs in the case of a detrainment process, which the jets experience much more rarely and in presence of peculiar situations, not always known.

**Figure 6:**
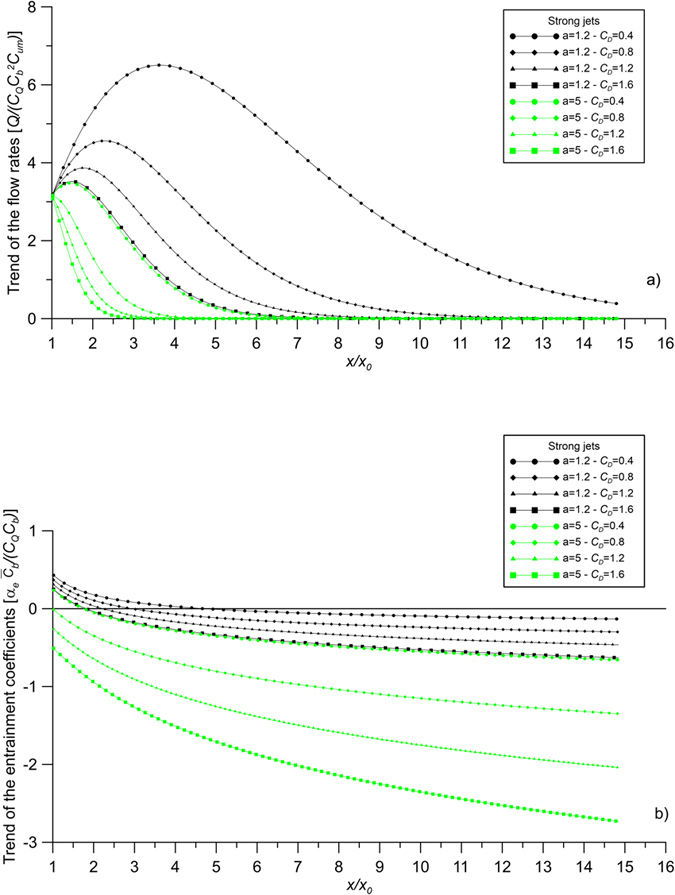
Trend of (**a**) the flow rates and (**b**) the entrainment coefficients of jets in obstructed current. Case of strong jets.

Figure 6a shows the trend of the flow rates of compound strong jets with obstructions, using eq. (32). Figure 6b shows the trend of the entrainment coefficients of compound strong jets using eq. (36). The figures show the values of the fully developed flow region, i.e. *x/x*_*0*_ > 1, where the analysis of the present paper is valid. The analogous values for the case of jets in unobstructed flows can be derived from eq. (36) assuming a nil bulk drag coefficient. As already written, the figures show a trend, since the coefficients of eqs (32) and (36) have been assumed of O(1), apart from *C*_*D*_ and a = *d*/*s*^2^. It is important to highlight that all these coefficients can be correctly evaluated using experimental data. The entrainment coefficient of a compound strong jet without obstructions has a positive value, which does not change with *x*, as shown by eq. (36) with a nil bulk drag coefficient. On the contrary, in some configurations of obstructed flows, which depend on the canopy characteristics, the entrainment coefficients have positive values in a limited zone, close to the jet origin, with, consequently, an increasing jet flow rate. Beyond this zone, the entrainment coefficients become negative, revealing a reduction of the jet flow rates (Fig. 6b). In other configurations the entrainment coefficients are always negative. When the entrainment coefficient is negative, the jet flow rate decreases, revealing a release of the transported solute towards the ambient fluid.

**Figure 7:**
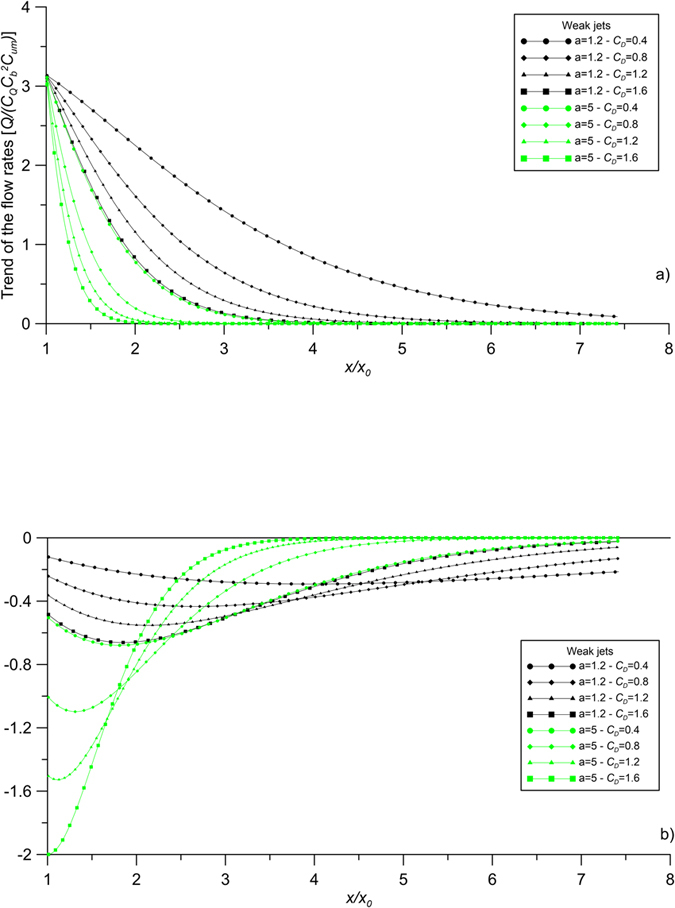
Trend of (**a**) the flow rates and (**b**) the entrainment coefficients of jets in obstructed current. Case of weak jets.

Figure 7a,b display the same results for the case of a compound weak jet. For the coefficients of eqs (37) and (39) the same hypothesis as the strong jets has been assumed. In any case, it is important to highlight that the curve trend, as well as the conclusions reported below, does not depend on the coefficient values. As shown by eq. (39), the entrainment coefficient of a compound jet in an unobstructed flow is nil, i.e. the jet flow rate is preserved. On the contrary, the same jet issued in an obstructed flow is characterized by a negative entrainment coefficient, with the consequence of a flow rate release towards the ambient fluid. Particularly, the entrainment coefficient reaches a minimum negative value at a certain value of *x/x*_*0*_, which depends on the canopy characteristics. The entrainment coefficient then starts to increase, remaining always negative. This conclusion is particularly interesting considering its effects on the ambient fluid, which experiences a release of the jet flow rate and, consequently, a release of its transported solute.

## Conclusions

This paper investigates the behavior of strong and weak compound jets, issued in obstructed flows, from a theoretical point of view. For both strong and weak jets issued in obstructed flows, the paper presents new equations of: (i) the flow rate; (ii) the entrainment coefficient. It is worth noting that these equations deeply depend on the bulk drag coefficient and canopy density. Some results have been also validated by laboratory experiments.

The obtained equations prove that a detrainment flow occurs when the jets are issued in obstructed flows, analogously to the cases, generally shown in literature, of jets released in stratified flows. The principal findings of our study can be summarized as follows.

1. In the case of a compound strong jet, the entrainment coefficient does not change with *x* in unobstructed flows. On the contrary, in some configurations of obstructed flows, which depend on the canopy characteristics, the entrainment coefficients have positive values in a limited zone, close to the jet origin, with, consequently, increasing flow rates of the jet. Beyond this zone, the entrainment coefficients become negative, revealing a reduction of the flow rates. In other configurations the entrainment coefficients are always negative. When the entrainment coefficient is negative, the jet flow rate decreases, revealing a release of the transported solute towards the ambient fluid.
2. In the case of a compound weak jet issued in an unobstructed flow, the entrainment coefficient is nil, as the bulk drag coefficient is nil, and consequently the jet flow rate is preserved. On the contrary, in obstructed flows, the jet flow rate decreases since the jet experiences a detrainment process. Specifically, the jet reaches the maximum detrainment coefficient at a specific value of *x/x*_*0*_. For greater values of *x/x*_*0*_, the detrainment coefficient increases, without becoming positive. This conclusion is particularly interesting, meaning that the ambient fluid experiences a release of the jet flow rate and, consequently, of its transported solute.

## Additional Information

**How to cite this article**: Mossa, M. and De Serio, F. Rethinking the process of detrainment: jets in obstructed natural flows. *Sci. Rep.* **6**, 39103; doi: 10.1038/srep39103 (2016).

**Publisher's note:** Springer Nature remains neutral with regard to jurisdictional claims in published maps and institutional affiliations.

## Supplementary Material

Supplementary Appendix 1

## Figures and Tables

**Figure 1:**
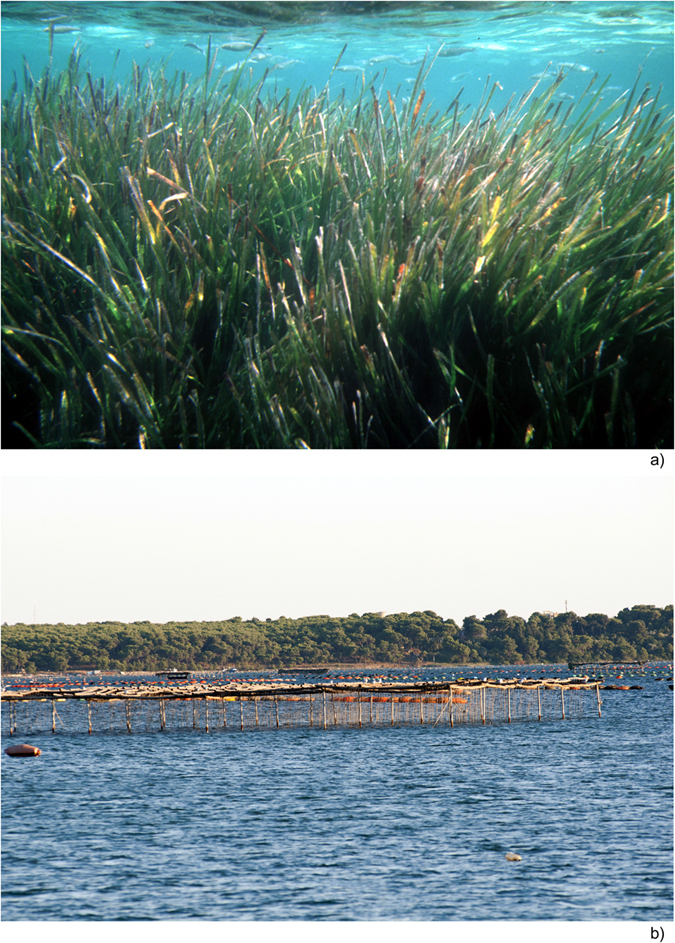
Example of canopies. (**a**) vegetated canopy; (**b**) mussel farms.

**Figure 2:**
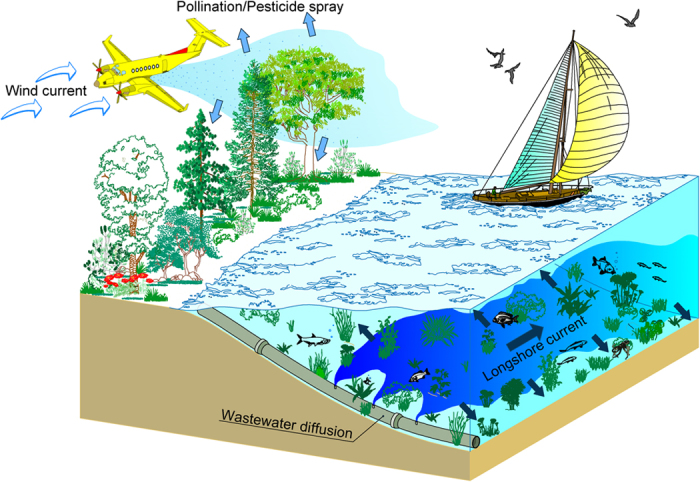
Typical release of jets in obstructed flows.

**Table 1:** Main parameters of the experimental Runs.

Runs	*H* [cm]	*U*_*e*_[ms^−1^]	*U*_*0*_[ms^−1^]	*U*_*0*_*/U*_*e*_ [−]	*Re* [−]	*Re*_*0*_[−]
1	37	0.16	5.90	0.027	23054	19904
2	30	0.19	5.90	0.032	26282	19904
3	37	0.16	3.93	0.041	24591	14154
4	30	0.19	3.93	0.050	26282	13270
