# Supplementary material for: Rethinking the process of detrainment: jets in obstructed natural flows
Source: Sci Rep. 2016 Dec 15;6:39103. doi: 10.1038/srep39103 (PMC5156915; doi:10.1038/srep39103)
Supplement: Supplementary Appendix 1 [file srep39103-s1.pdf]

Rethinking the process of detrainment: jets in obstructed natural flows

Michele Mossa* and Francesca De Serio

Technical University of Bari, DICATECh - Department of Civil, Environmental, Building Engineering and Chemistry
Via E. Orabona 4 - 70125 Bari, Italy
email: michele.mossa@poliba.it; francesca.deserio@poliba.it
*corresponding author

Appendix 1

Complete equation analysis of a plane turbulent jet issued in still obstructed water

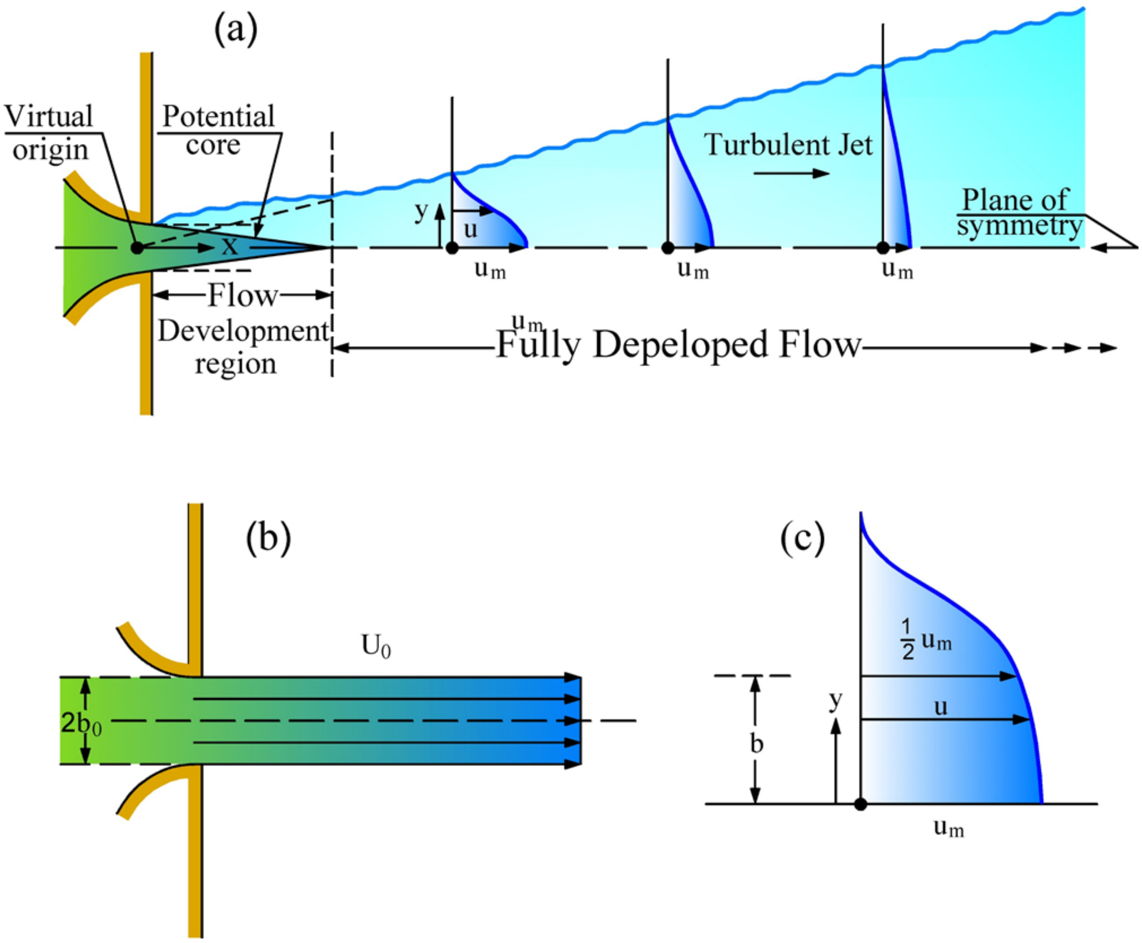

Figure A1: Sketch of plane jet.

In the case of a plane turbulent jet issued in an ambient fluid at rest with the presence of a cylinder array (figure A1), the Reynolds equations of motions are (A1), where *u*, *v*, and *w* and *u'*, *v'* and *w'* are the time-averaged and fluctuating velocity in the *x*, *y*, and *z* directions, respectively, *p* is the time-averaged pressure at any point, ν is the kinematic viscosity, ρ is the mass density of the jet and ambient fluid and t is the time variable.

$$\frac{\partial u}{\partial t}+u\frac{\partial u}{\partial x}+v\frac{\partial u}{\partial y}+w\frac{\partial u}{\partial z}=-\frac{1}{\rho}\frac{\partial p}{\partial x}+\nu\left(\frac{\partial^2 u}{\partial x^2}+\frac{\partial^2 u}{\partial y^2}+\frac{\partial^2 u}{\partial z^2}\right)-\left(\frac{\partial \overline{u'^2}}{\partial x}+\frac{\partial \overline{u'v'}}{\partial y}+\frac{\partial \overline{u'w'}}{\partial z}\right)-\frac{1}{\rho}D_x$$

$$\frac{\partial v}{\partial t}+u\frac{\partial v}{\partial x}+v\frac{\partial v}{\partial y}+w\frac{\partial v}{\partial z}=-\frac{1}{\rho}\frac{\partial p}{\partial y}+\nu\left(\frac{\partial^2 v}{\partial x^2}+\frac{\partial^2 v}{\partial y^2}+\frac{\partial^2 v}{\partial z^2}\right)-\left(\frac{\partial \overline{u'v'}}{\partial x}+\frac{\partial \overline{v'^2}}{\partial y}+\frac{\partial \overline{v'w'}}{\partial z}\right)-\frac{1}{\rho}D_y \quad \text{(A1)}$$

$$\frac{\partial w}{\partial t}+u\frac{\partial w}{\partial x}+v\frac{\partial w}{\partial y}+w\frac{\partial w}{\partial z}=-\frac{1}{\rho}\frac{\partial p}{\partial z}+\nu\left(\frac{\partial^2 w}{\partial x^2}+\frac{\partial^2 w}{\partial y^2}+\frac{\partial^2 w}{\partial z^2}\right)-\left(\frac{\partial \overline{u'w'}}{\partial x}+\frac{\partial \overline{v'w'}}{\partial y}+\frac{\partial \overline{w'^2}}{\partial z}\right)-\frac{1}{\rho}D_z$$

The drag force in the *x*, *y* and *z* direction, i.e. the resistance due to the solid medium, sum of form and viscous drag over the stem are D_x, D_y and D_z, respectively. As shown by Nepf[34], various resistance laws for flow in porous media can be derived. Particularly, in open channel or atmospheric vegetated flow, the quadratic form

$$D_i=\frac{1}{2}\rho C_D a|u_i|u_i \quad \text{(A2)}$$

with *i=x,y,z.* The continuity equation is

$$\frac{\partial u}{\partial x}+\frac{\partial v}{\partial y}+\frac{\partial w}{\partial z}=0 \quad \text{(A3)}$$

Since the flow is quasi-two dimensional we can approximately assume that *w*=0, ∂/∂z =0, $\overline{u'w'}$=0 and $\overline{v'w'}=0$. Considering that the mean flow is steady ∂/∂t =0. Furthermore, *u* is generally much larger than *v* in a large portion of the jet and velocity and stress gradients in the y-direction are much larger. Therefore, the equations (A1) become

$$u\frac{\partial u}{\partial x}+v\frac{\partial u}{\partial y}=-\frac{1}{\rho}\frac{\partial p}{\partial x}+\nu\frac{\partial^2 u}{\partial y^2}-\frac{\partial \overline{u'^2}}{\partial x}-\frac{\partial \overline{u'v'}}{\partial y}-\frac{1}{\rho}D_x \quad \text{(A4)}$$

$$0=-\frac{1}{\rho}\frac{\partial p}{\partial y}-\frac{\partial \overline{v'^2}}{\partial y}-\frac{1}{\rho}D_y \quad \text{(A5)}$$

Integrating (A5), assuming p_∞ as the pressure outside the jet we get

$$p=p_\infty-\rho\overline{v'^2}-\int_0^\infty D_y dy \quad \text{(A6)}$$

Differentiating eq. (A6), we obtain

$$u\frac{\partial u}{\partial x}+v\frac{\partial u}{\partial y}=-\frac{1}{\rho}\frac{dp_\infty}{dx}+\nu\frac{\partial^2 u}{\partial y^2}-\frac{\partial \overline{u'v'}}{\partial y}-\frac{\partial}{\partial x}\left(\overline{u'^2}-\overline{v'^2}\right)-\frac{1}{\rho}\left(D_x+\frac{d}{dx}\int_0^\infty D_y dy\right) \quad \text{(A7)}$$

where generally it is reasonable assuming that

$$D_x >> \frac{d}{dx}\int_0^\infty D_y dy \tag{A8}$$

and the penultimate term of eq. (A7) is smaller than the other terms and could be dropped. Therefore, we get

$$u\frac{\partial u}{\partial x}+v\frac{\partial u}{\partial y}=-\frac{1}{\rho}\frac{dp_\infty}{dx}+\nu\frac{\partial^2 u}{\partial y^2}-\frac{\partial\overline{u'v'}}{\partial y}-\frac{1}{\rho}D_x \tag{A9}$$

Observing that the laminar and turbulent stresses are respectively

$$\begin{cases}\tau_l=\mu\dfrac{\partial u}{\partial y}\\ \tau_t=-\rho\overline{u'v'}\end{cases} \tag{A10}$$

and considering that τ_t is much larger than τ_l and that generally the pressure gradient in the longitudinal direction is smaller than the other terms, we get

$$u\frac{\partial u}{\partial x}+v\frac{\partial u}{\partial y}=\frac{1}{\rho}\frac{\partial \tau_t}{\partial y}-\frac{1}{\rho}D_x \tag{A11}$$

For the sake of brevity, below τ_t will be written as τ. Integrating the above equation, we get

$$\rho\int_0^\infty u\frac{\partial u}{\partial x}dy+\rho\int_0^\infty v\frac{\partial u}{\partial y}dy-\int_0^\infty\frac{\partial\tau}{\partial y}dy=-\int_0^\infty D_x dy \tag{A12}$$

that becomes

$$\frac{1}{2}\frac{d}{dx}\int_0^\infty \rho u^2 dy+\rho\left(\left|uv\right|_0^\infty-\int_0^\infty u\frac{\partial v}{\partial y}dy\right)=-\int_0^\infty D_x dy \tag{A13}$$

Using the continuity eq. (A3), we get

$$\frac{d}{dx}\int_0^\infty \rho u^2 dy=-\int_0^\infty D_x dy \tag{A14}$$

Assuming that

$$D_x=\frac{1}{2}\rho C_D a u^2 \tag{A15}$$

it possible to obtain that

$$\frac{dM\left(x\right)}{dx}=\frac{d}{dx}\int_0^\infty \rho u^2 dy=-\frac{1}{2}C_D a\int_0^\infty \rho u^2 dy \tag{A16}$$

whose solution is

$$M(x) = M_0 \exp\left(-\frac{1}{2}C_D ax\right) \tag{A17}$$

Equation (A17) shows an interesting result, since a pure plane jet in an obstructed flow does not preserve the momentum as in the analogous case of unobstructed flow.

For the similarity analysis assuming that $\eta = y/b$ we get

$$\frac{u}{u_m} = f(\eta). \tag{A18}$$

Furthermore, assuming simple forms for u_m and b

$$\begin{cases} u_m \propto x^p \\ b \propto x^q \end{cases} \tag{A19}$$

it is possible to write the following equation

$$\frac{d}{dx}\int_0^\infty \rho u_m^2 f^2 b d\eta = -\frac{1}{2}C_D a\int_0^\infty \rho u_m^2 f^2 b d\eta \tag{A20}$$

i.e.

$$\frac{d}{dx}\left(\rho u_m^2 b\int_0^\infty f^2 d\eta\right) = -\frac{1}{2}C_D a\int_0^\infty \rho u_m^2 f^2 b d\eta \tag{A21}$$

Considering that

$$\int_0^\infty f^2 d\eta \tag{A22}$$

is constant with x, eq. (A21) becomes

$$\frac{d}{dx}\left(u_m^2 b\right) = -\frac{1}{2}C_D abu_m^2 \tag{A23}$$

i.e.

$$2p + q = -\frac{1}{2}C_D ax \tag{A24}$$

From dimensional considerations, it is possible to write that

$$\frac{\tau}{\rho u_m^2} = g(\eta) \tag{A25}$$

Considering eqs. (A18) and (A25), assuming

$$\begin{cases} f' = \dfrac{df}{d\eta} \\ g' = \dfrac{dg}{d\eta} \\ b' = \dfrac{db}{dx} \\ u'_m = \dfrac{du_m}{dx} \end{cases} \tag{A26}$$

we get

$$u\frac{\partial u}{\partial x} = u_m u'_m\, f^2 - \frac{u_m^2 b'}{b}\eta ff' \tag{A27}$$

$$v\frac{\partial u}{\partial y} = \frac{u_m^2 b'}{b}\left(\eta ff' - f'\int_0^\eta fd\eta\right) - u_m u'_m\, f'\int_0^\eta fd\eta \tag{A28}$$

$$\frac{1}{\rho}\frac{\partial \tau}{\partial y} = \frac{u_m^2}{b} g' \tag{A29}$$

and

$$-\frac{1}{\rho}D_x = -\frac{1}{2}C_D a u_m^2 f^2 \tag{A30}$$

Substituting (A27), (A28), (A29) and (A30) into (A11), it is possible to obtain

$$g' = \frac{bu'_m}{u_m}\left(f^2 - f'\int_0^\eta fd\eta\right) - b'\left(\eta f' - \eta ff' + f'\int_0^\eta fd\eta\right) + \frac{1}{2}C_D abf^2. \tag{A31}$$

Since *g'* is a function of only η, also the right-hand side should be a function of only η. Particularly, from the first two terms on the right-hand side, it is possible to write

$$\begin{cases} \dfrac{bu'_m}{u_m} \propto x^{q-1} \\ b' \propto x^{q-1} \end{cases} \tag{A32}$$

and, therefore,

$$q = 1 \tag{A33}$$

i.e.

$$b = C_1 \cdot x \tag{A34}$$

In the present study, we will consider jets with

$$b = O(n \cdot s) \tag{A35}$$

with n=O(10-100). In other words we are considering the case where d/s=O(10^{-1}-1), b/d=O(10-10^2), b/s=O(10-10^2), such as that shown in figure 4.

Therefore, it is possible to write

$$b = C_1 x^q = O\left(\left(10 \div 100\right) \cdot s\right) \tag{A36}$$

The order of magnitude of *b* changes when it becomes

$$10b = 10C_1 x^q = O\left(\left(100 \div 1000\right) \cdot s\right) \tag{A37}$$

i.e. when x^q increases of an order of magnitude or more. Therefore, *b* has the same order of magnitude between two values of *x*, i.e. from x_1 to $x_2 > x_1$, when

$$\frac{x_2^q}{x_1^q} < O(10) \tag{A38}$$

Since in the analyzed case *q*=1 it is possible to write that

$$x_1, x_2 = O(n \cdot s) \text{ with } n \geq 10-100 \tag{A39}$$

Therefore, along a longitudinal distance between x_1 and x_2 satisfying eq. (A39), eq. (A31) becomes

$$g' \approx \frac{bu'_m}{u_m}\left(f^2 - f'\int_0^\eta f d\eta\right) - b'\left(\eta f' - \eta f f' + f'\int_0^\eta f d\eta\right) + \frac{1}{2} C_D \frac{d}{s^2} n s f^2 \tag{A40}$$

where the last term can be considered approximately constant in the limits above described.

With these considerations in mind and using eqs. (A32) and (A24), it is possible to conclude that

$$\begin{cases} q = O(1) \\ p = O\left(-\frac{1}{2}\right) - \frac{1}{4} C_D a x = O\left(-\frac{1}{2}\right) - \frac{1}{4} C_D \frac{d}{s^2} x \end{cases} \tag{A41}$$

Assume

$$b = C_b \left(\frac{x}{x_0}\right)^q \tag{A42}$$

$$u_m = C_{um} \left(\frac{x}{x_0}\right)^p \tag{A43}$$

where x_0 is the distance from the nozzle where the jet flow starts to be fully developed and C_b and C_{um} are the values of *b* and u_m, respectively, for *x* equal to x_0. The jet flow rate per unit depth becomes equal to

$$Q \propto 2b \cdot u_m \Rightarrow Q = 2C_Q C_b C_{um} \frac{x}{x_0} \cdot \left(\frac{x}{x_0}\right)^{-\frac{1}{2}-\frac{1}{4}C_D a x} = 2C_Q C_b C_{um} \left(\frac{x}{x_0}\right)^{\frac{1}{2}-\frac{1}{4}C_D a x} \tag{A44}$$

where C_Q is a dimensionless coefficient which considers both the geometry of each cross section of the jet and the ratio between the average longitudinal velocity and the maximum longitudinal velocity of each analyzed cross section. In other word the volume flow rate in each cross section of the jet is equal to

$$Q = 2\bar{b} \cdot U_b \tag{A45}$$

where $\overline{b}$ is the nominal outer boundary of the jet where u is close to zero and U_b is the average longitudinal velocity in the analyzed cross section of the jet. Therefore, from eqs. (A44) and (A45) we get

$$C_Q = \frac{\overline{b}}{b} \cdot \frac{U_b}{u_m} \tag{A46}$$

Considering the entrainment coefficient of the jet α_e, we derive that

$$\frac{dQ}{dx} = 2v_e = 2u_m\alpha_e \tag{A47}$$

where v_e is the transversal velocity at the nominal outer boundary of the jet, which is oriented towards the jet centerline in the case of a positive entrainment coefficient and vice versa in the case of a negative entrainment coefficient, i.e. when a detrainment flow is present. Therefore

$$\frac{dQ}{dx} = -\frac{C_Q C_b C_{um}}{2s^2 x} \cdot \left(\frac{x}{x_0}\right)^{\frac{1}{2}-\frac{C_D dx}{4s^2}} \left(C_D dx + C_D dx \ln\left(\frac{x}{x_0}\right) - 2s^2\right) \tag{A48}$$

and

$$\alpha_e = \frac{C_Q C_b}{4x_0} \cdot \left(2 - C_D ax - C_D ax \ln\left(\frac{x}{x_0}\right)\right) \tag{A49}$$
